# Platypus: an open-access software for integrating lymphocyte single-cell immune repertoires with transcriptomes

**DOI:** 10.1093/nargab/lqab023

**Published:** 2021-04-14

**Authors:** Alexander Yermanos, Andreas Agrafiotis, Raphael Kuhn, Damiano Robbiani, Josephine Yates, Chrysa Papadopoulou, Jiami Han, Ioana Sandu, Cédric Weber, Florian Bieberich, Rodrigo Vazquez-Lombardi, Andreas Dounas, Daniel Neumeier, Annette Oxenius, Sai T Reddy

**Affiliations:** Department of Biosystems Science and Engineering, ETH Zurich, 4058 Basel, Switzerland; Institute of Microbiology, ETH Zurich, 8093 Zurich, Switzerland; Department of Pathology and Immunology, University of Geneva, 1211 Geneva, Switzerland; Department of Biosystems Science and Engineering, ETH Zurich, 4058 Basel, Switzerland; Department of Biosystems Science and Engineering, ETH Zurich, 4058 Basel, Switzerland; Department of Biosystems Science and Engineering, ETH Zurich, 4058 Basel, Switzerland; Department of Biosystems Science and Engineering, ETH Zurich, 4058 Basel, Switzerland; Department of Biosystems Science and Engineering, ETH Zurich, 4058 Basel, Switzerland; Department of Biosystems Science and Engineering, ETH Zurich, 4058 Basel, Switzerland; Institute of Microbiology, ETH Zurich, 8093 Zurich, Switzerland; Department of Biosystems Science and Engineering, ETH Zurich, 4058 Basel, Switzerland; Department of Biosystems Science and Engineering, ETH Zurich, 4058 Basel, Switzerland; Department of Biosystems Science and Engineering, ETH Zurich, 4058 Basel, Switzerland; Institute for Biomedical Engineering, University and ETH Zurich, 8092 Zurich, Switzerland; Department of Biosystems Science and Engineering, ETH Zurich, 4058 Basel, Switzerland; Institute of Microbiology, ETH Zurich, 8093 Zurich, Switzerland; Department of Biosystems Science and Engineering, ETH Zurich, 4058 Basel, Switzerland

## Abstract

High-throughput single-cell sequencing (scSeq) technologies are revolutionizing the ability to molecularly profile B and T lymphocytes by offering the opportunity to simultaneously obtain information on adaptive immune receptor repertoires (VDJ repertoires) and transcriptomes. An integrated quantification of immune repertoire parameters, such as germline gene usage, clonal expansion, somatic hypermutation and transcriptional states opens up new possibilities for the high-resolution analysis of lymphocytes and the inference of antigen-specificity. While multiple tools now exist to investigate gene expression profiles from scSeq of transcriptomes, there is a lack of software dedicated to single-cell immune repertoires. Here, we present Platypus, an open-source software platform providing a user-friendly interface to investigate B-cell receptor and T-cell receptor repertoires from scSeq experiments. Platypus provides a framework to automate and ease the analysis of single-cell immune repertoires while also incorporating transcriptional information involving unsupervised clustering, gene expression and gene ontology. To showcase the capabilities of Platypus, we use it to analyze and visualize single-cell immune repertoires and transcriptomes from B and T cells from convalescent COVID-19 patients, revealing unique insight into the repertoire features and transcriptional profiles of clonally expanded lymphocytes. Platypus will expedite progress by facilitating the analysis of single-cell immune repertoire and transcriptome sequencing.

## INTRODUCTION

Immune repertoires are comprised of a diverse collection of B-cell receptors (BCRs) and T-cell receptors (TCRs), which enable molecular recognition to a vast number of pathogen and disease antigens. Immune repertoire diversity is initially generated as a result of lymphocyte V(D)J recombination and, in the case of B cells, can undergo further sequence diversification in the form of somatic hypermutation. Targeted deep sequencing of BCRs and TCRs from bulk populations of lymphocytes has paved the way to quantify the diversity, distribution and evolution of immune repertoires (1–4). However, one major challenge in immune repertoire sequencing is acquiring information on correct receptor chain pairing [variable light (VL) and variable heavy (VH) for BCR and variable alpha (Vα) and variable beta (Vβ) for TCR], which greatly complicates identification of clonal groups and antigen-specificity (5,6). Until only recently it was not possible to directly integrate a lymphocyte's phenotypic gene expression information (i.e. activation, exhaustion and antibody secretion) with its immune receptor sequence.

Recent developments in microfluidic and scSeq technologies have now made it possible to obtain information on immune repertoires or transcriptional profiles at high-throughput (7–9). Several of these methods have been tailored specifically for lymphocytes, thus making it possible to perform parallel sequencing of immune repertoires and whole transcriptomes (10,11). Furthermore, commercially available instruments and protocols [10× Genomics Chromium and VDJ and gene expression (GEX) libraries] are further accelerating progress in this field. This simultaneous sequencing of immune repertoires and transcriptomes produces single-cell datasets with features ranging from quantitative gene profiles, cellular phenotypes, transcriptional clustering, clonal diversity and expansion, germline gene usage, somatic hypermutation, among many others. These high-dimensional datasets can be mined to discover novel insight on lymphocyte immunobiology, function and specificity. For example, one recent study leveraged scSeq to discover the distinct transcriptional profiles and specificity of B cells following influenza vaccination (8). In another study, clonal expansion and activation signatures of tumor-infiltrating T cells were profiled (12). Other studies have leveraged this technology to answer fundamental questions across a variety of areas in immunology such as bacterial infection responses (13), tumor-immune microenvironment (14), clonal expansion in Alzheimer's disease (9) and B-cell differentiation (15).

While multiple bioinformatic tools exist to facilitate rapid analysis of gene expression from scSeq (16–19), they do not allow the incorporation of immune repertoire information. Analogously, existing software packages to analyze immune repertoires do not allow the user to supply accompanying gene expression and transcriptome data (20–22). Taken together, these considerations complicate the analysis of BCR and TCR repertoires for those with little bioinformatics experience and who are unfamiliar with the output data from scSeq experiments. To address the lack of software specifically tailored to single-cell lymphocyte sequencing data, we developed Platypus, an open-source R package that contains an automated pipeline to analyze and integrate single-cell immune repertoires with transcriptome data. With only a few lines of code, Platypus allows users to easily extract immune repertoire features such as clonal expansion, somatic hypermutation, isotype switching and integrate it with transcriptome features such as differential gene expression. We subsequently demonstrate the value of this package using scSeq data from convalescent coronavirus disease 2019 (COVID-19) patients. Our analysis revealed clonal expansion in B and T cells, and within these we could identify distinct patterns of somatic hypermutation, amino acid usage, clonal convergence and transcriptional heterogeneity. Taken together, Platypus helps facilitate the analysis of single cell immune repertoires and transcriptomes and reveal novel insights such as the transcriptional profile of clonal expanded and potentially pathogen-reactive lymphocytes.

## MATERIALS AND METHODS

### Patient samples

Patients were participants of the SERO-BL-COVID-19 study sponsored by the Department of Health, Canton Basel-Landschaft, Switzerland. Both patients tested positive for SARS-CoV-2 after reverse transcription polymerase chain reaction (RT-PCR) of naso- and oropharyngeal swab and did not require hospitalization. Whole blood was collected 31 and 32 days following a positive RT-PCR test and subjected to density gradient centrifugation using the Ficoll Paque Plus reagent (GE Healthcare, #17-1440-02). After separation, the upper plasma layer was collected for ELISA detection of IgG and IgA SARS-CoV-2-specific antibodies (Euroimmun Medizinische Labordiagnostika, #EI2668-9601G, #EI2606-9601A). Peripheral blood mononuclear cells (PBMC) were collected from the interphase, resuspended in freezing medium (Roswell Park Memorial Institute (RPMI) medium 1640, 10%(v/v) fetal bovine serum (FBS), 10%(v/v) dimethyl sulfoxide) and cryopreserved in liquid nitrogen. Point-of-care lateral flow immunoassays assessing the presence of IgG and IgM SARS-CoV-2-specific antibodies (Qingdao Hightop Biotech, #H100) were performed at the time of blood collection.

### Immunomagnetic isolation of B and T cells

PBMC samples were thawed, washed in complete media (RPMI 1640, 10%(v/v) FBS) and pelleted by centrifugation. Cells were resuspended in 0.5 ml complete media, counted and treated with 10 U ml^–1^ DNase I (Stemcell Technologies, #07900) for 15 min at RT in order to prevent cell clumping. After DNase I digestion, cells were washed in complete media, pelleted by centrifugation and resuspended in 0.5 ml flow cytometry buffer [phosphate-buffered saline (PBS), 2%(v/v) FBS, 2 mM ethylenediaminetetraacetic acid]. The cell suspension was filtered through a 40 μM cell strainer prior to immunomagnetic isolation. As a first step, plasma cells were isolated using the EasySep Human CD138 Positive Selection Kit II for future studies (Stemcell Technologies, #17877). The negative fraction of the above selections was divided into two aliquots that were subjected to negative immunomagnetic isolation of either B cells (EasySep Human Pan-B-cell Enrichment Kit, Stemcell Technologies, #19554) or T cells (EasySep Human T cell Isolation Kit, Stemcell Technologies, #17951). After isolation, B and T cells were pelleted by centrifugation, resuspended in PBS, 0.4% bovine serum albumin (BSA)(v/v), filtered through a 40 μM cell strainer and counted. T and B cells originating from the same patient were pooled in equal numbers and the final suspension was counted and assessed for viability using a fluorescent cell counter (Cellometer Spectrum, Nexcelom). Whenever possible, cells were adjusted to a concentration of 1 × 10^6^ live cells/ml in PBS, 0.04%(v/v) BSA before proceeding with droplet generation.

### Single cell sequencing libraries

Single cell 10× libraries were constructed from the isolated single cells following the Chromium Single Cell V(D)J Reagent Kits User Guide (CG000086 Rev M). Briefly, single cells were co-encapsulated with gel beads (10× Genomics, 1000006) in droplets using four lanes of one Chromium Single Cell A Chip (10× Genomics, 1000009). V(D)J library construction was carried out using the Chromium Single Cell 5’ Library Kit (10× Genomics, 1000006) and the Chromium Single Cell V(D)J Enrichment Kit, Human B Cell (10× Genomics) and Human T Cell (10× Genomics). The reverse transcribed cDNA was split in three and GEX, B and T cell V(D)J libraries were constructed following the instructions from the manufacturer. Final V(D)J libraries were pooled and sequenced on the Illumina NovaSeq platform (300 cycles, paired-end reads). Pooled 5’ gene expression libraries were and sequenced on the Illumina NextSeq 500 (26/91 cycles, paired-end) with a concentration of 1.6 pM with 1% PhiX. Resulting FASTQ files were demultiplexed and subsequently used as input to cellranger (v3.1.0, 10× Genomics). GEX sequencing libraries were aligned to the refdata-cellranger-GRCh38–3.0.0 reference genome and VDJ genes and the VDJ sequencing libraries were aligned to the vdj_GRCh38_alts_ensembl-3.1.0–3.1.0 reference using Single Cell V(D)J R2-only chemistry.

### Immune repertoire analysis using Platypus

The R package, accompanying code, and processed sequencing data used in this study are publicly available at github.com/alexyermanos/Platypus and doi.org/10.5281/zenodo.4140161. Briefly, clonotyping information was extracted directly from the output directory of cellranger using the function analyze_VDJ in Platypus (v2.0.3). Quantifying the number of unique clones was performed using the VDJ_clonotype function in Platypus, with clone.strategy set to either ‘cdr3.aa’, ‘hvj.lvj’, ‘hvj.lvj.cdr3length.cdr3homology’, or ‘hvj.lvj.cdr3lengths’. The isotype distributions for the top thirty B-cell clones were calculated using the VDJ_isotypes_per_clone function in Platypus. CDR3 length distribution and sequence logo plots were calculated on the output of the VDJ_per_clone function. Sequence logos were calculated based on the the R package ggseqlogo (v0.1) (32), with method set to ‘prob’ and seq_type set to ‘aa’. The output directory from cellranger count was supplied as input to the function automate_GEX, that analyzes and integrates transcription data using functions from the R package Seurat (v3.1.1). Briefly, the GEX libraries were integrated using the SCTransform function from Seurat. Cells containing more than 20% mitochondrial genes were removed. TCR and BCR genes were filtered prior to integration and gene expression analysis. The number of variable features selected was 2000 for the RunPCA function using the first 10 dimensions and cluster resolution was set to the default 0.5. Transcriptional cluster and clonotype information were integrated using the VDJ_GEX_integrate function in Platypus under default parameters. Quantification of the transcriptional cluster distribution for the 10 most expanded clones from patient 1 were calculated and visualized using the VDJ_GEX_expansion function in Platypus. Those cells from the two most expanded clones containing barcodes in both VDJ and GEX datasets were highlighted on the UMAP plot using the function visualize_clones_GEX in Platypus under default parameters. Somatic hypermutation was calculated in the VDJRegion as defined by MiXCR via the function call_MIXCR in Platypus, which utilized MiXCR (v3.0.12) (22). The number of nucleotide alignment mismatches between the reference germline and the full-length VDJRegion for both heavy and light chain nucleotide sequence was then computed based on the best alignment determined by MIXCR. The phylogenetic tree was inferred by appending the full-length VDJRegion of the heavy and light chain for each unique sequence after appending V_H_ VDJRegion and V_L_ VDJRegion, as determined from the output function of call_MIXCR in Platypus. The reference germline sequence was first extracted from the initial cellranger alignment using the function VDJ_extract_germline in Platypus and added to the set of input sequences which were supplied to VDJ_tree. Sequence similarity networks were calculated using the function VDJ_networks in Platypus by calculating the edit distance separately for CDRH3 and CDRL3 amino acid sequences and then summing the two matrices. Edges were then drawn between those clones separated by less than either 14 or 10 amino acid mutations. Networks from the VDJ_network function in Platypus relied upon igraph (v1.2.4.1). The heatmap integrating clonotype membership with user-defined gene lists was created using the GEX_heatmap function in Platypus. Additional R packages utilized by Platypus in this study include ggplot2 (v3.2.1), stringdist (v0.9.5.5), stringr (v1.4.0), dplyr (v1.0.1), seqinr (v3.6.1), org.Mm.eg.db_3.10.0, scales (v1.0.0) and knitr (v1.28). All analysis was performed using R (v3.6.1) within Rstudio (v1.2.5019) using a MacBook Pro (2016, v10.14.16) and could be reproduced on Windows 10 Pro (v1909) using R (v4.0.0) within Rstudio (v1.1.463).

Circos plots depicting V-J gene usage were produced the VDJ_VJ_usage_circos function in Platypus for the top 10 clones with the c.threshold = 1, label.threshold = 50 and cell.level = T arguments. This function relies upon the circlize R package (v0.4.12) (33). V-J gene usage heatmaps were produced using the pheatmap package (v1.0.12). Gene ontology was performed using the GEX_GOterm function in Platypus under default parameters, which relies upon functions from edgeR (v3.28.1) (34) and limma (v3.42.2) (35). Gene set enrichment analysis was performed using the GEX_GSEA function in Platypus under default parameters, which additionally relies upon fgsea (v1.12) (36), tibble (v2.1.3) and the C7 gene set from the molecular signatures database (37) (MSigDB, gsea-msigdb.org/gsea/msigdb/collections.jsp#C7).

## RESULTS

### Single-cell immune repertoire sequencing analysis

**Figure 1.**
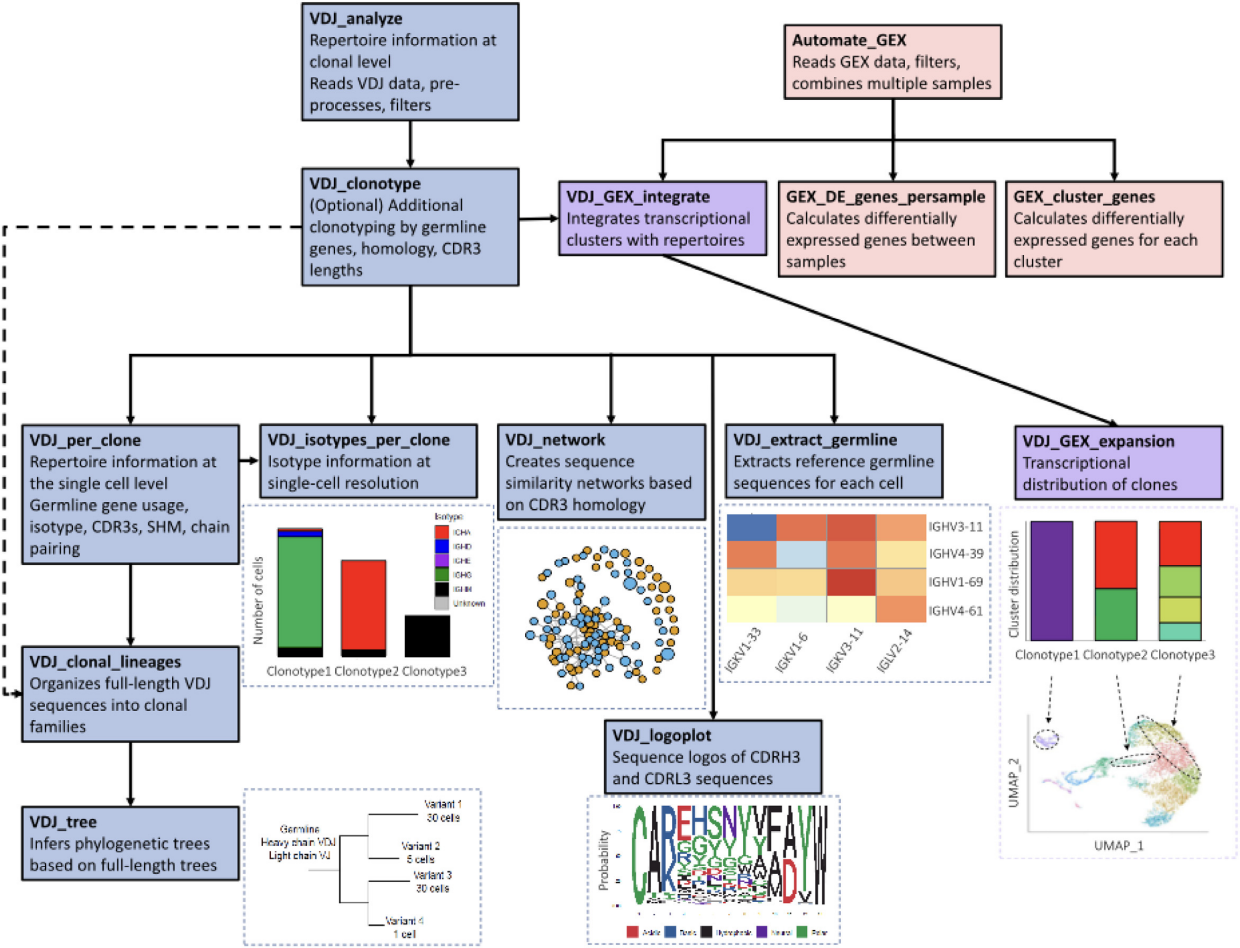
Flowchart demonstrating the workflow of Platypus. A selection of functions internal to Platypus and their respective relationships are depicted. Each node in the flow chart indicates a process in the workflow requiring just a single line of code with user-definable parameters.

Platypus allows the user to integrate single-cell immune repertoire and transcriptome sequencing data, which includes automation of pre-processing, filtering and data visualization (Figure 1). While Platypus is optimized for data generated by the 10× Genomics System, it is also adaptable to other cell-barcode based scSeq data (e.g. RAGE-seq, Split-Seq (10,23)). Users can supply the path to the output directory from the 10× cellranger alignment as input to Platypus, which then extracts and annotates key immune repertoire metrics such as clonal diversity, clonal expansion, somatic hypermutation, reference germline gene usage and sequence motifs (Figure 1). Platypus can perform additional clonotyping, either increasing or relaxing the pre-determined stringency of upstream alignment tools by incorporating information regarding germline gene usage or sequence homology thresholds. In addition to clonal sequence information [based on complementarity determining region 3 (CDR3)], it also extracts full-length sequences of both immune receptor variable chains (V_H_ and V_L_ for BCRs and Vα and Vβ for TCRs). Furthermore, Platypus enables the quantification, comparison and visualization of more advanced repertoire features such as sequence similarity networks (1), phylogenetic tree construction (24,25), isotype quantification and diversity metrics (26).

**Figure 2.**
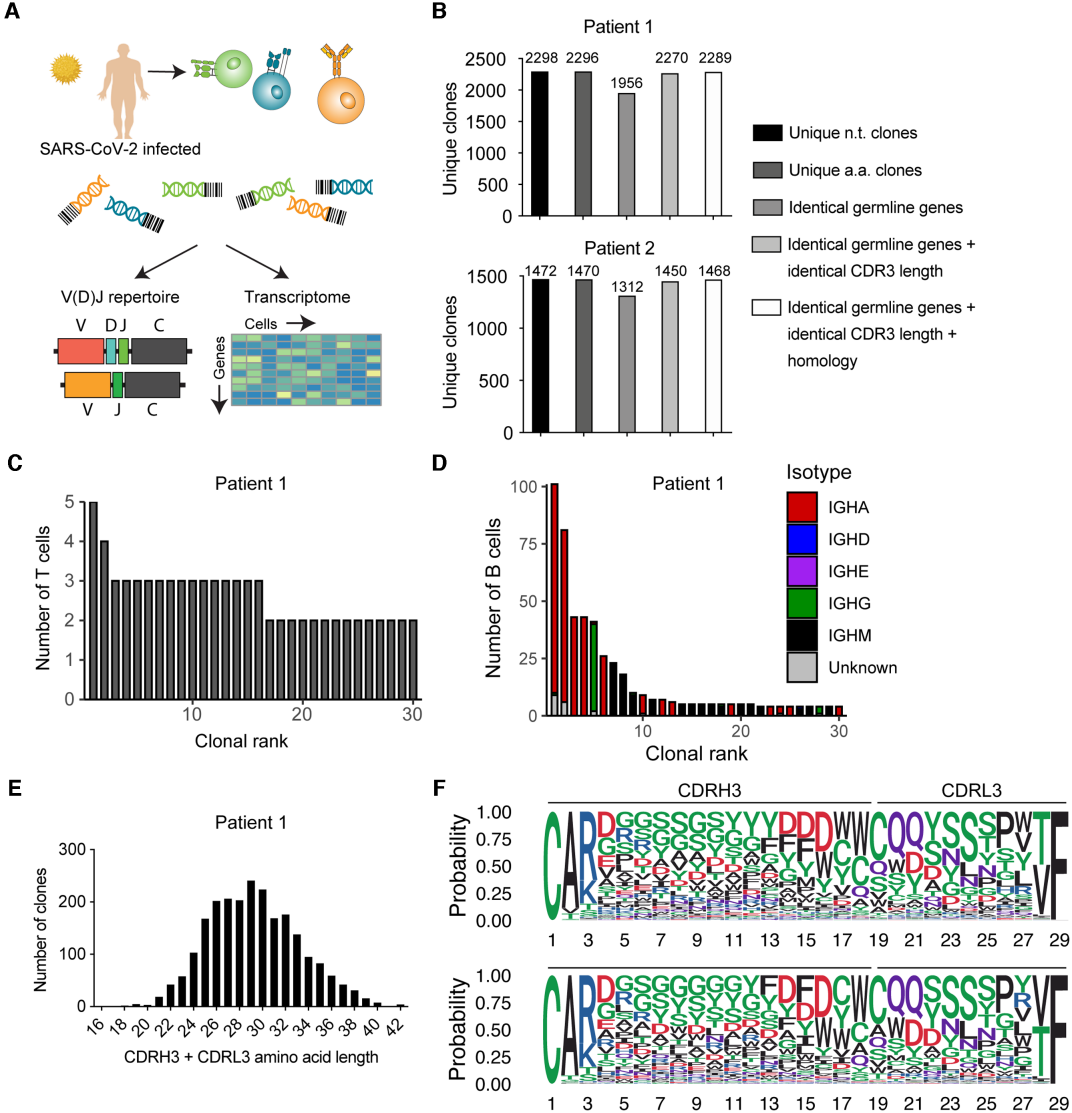
Extracting and visualizing clonal information of PBMCs from patients recently infected with SARS-CoV-2 using Platypus. (**A**) Experimental overview for single-cell immune repertoire of B and T cells in two patients previously infected with SARS-CoV-2. (**B**) Multiple B-cell clonotyping strategies involving CDR3 sequence identity, germline gene usage and sequence homology thresholds from the two patients previously infected with SARS-CoV-2. (**C**) Clonal expansion profiles of the T cells from the blood repertoires of one individual recently infected with SARS-CoV-2. Clone is defined as unique CDRβ3 + CDRα3 nucleotide sequence. (**D**) Clonal expansion profiles of the B cells from the blood repertoires of one individual recently infected with SARS-CoV-2. Clone is defined by unique CDRH3 + CDRL3 nucleotide sequence. Color depicts isotype on the cell level within each clone determined by the VDJ repertoire sequencing libraries. Plots produced using VDJ_isotypes_per_clone in Platypus. (**E**) Length distribution of the paired CDRH3 + CDRL3 amino acid sequences from the B-cell clones of a single patient. (**F**) Sequence logo plots of those paired CDRH3 + CDRL3 amino acid sequences with a combined sequence length of 29 in two patients. Colors correspond to biochemical properties: red = acidic, blue = basic, black = hydrophobic, purple = neutral, green = polar. Top logo plot corresponds to patient 1 and bottom logo plot corresponds to patient 2.

To demonstrate the potential of Platypus, we performed single-cell immune repertoire and transcriptome sequencing on B and T cells isolated from PBMCs of two convalescent COVID-19 patients (Figure 2A). 10× Genomics’ basic alignment tool is cellranger, which has a default clonotyping strategy that groups identical CDRH3 + CDRL3 nucleotide sequences into clonal families; this approach would be too restrictive in identifying clonotypes of B cells that have undergone somatic hypermutation in the CDR3s. We thereby demonstrated the ability and impact of changing the clonotyping strategy to include germline genes, CDR3 length restrictions and sequence homology requirements for the B-cell repertoires of the two COVID-19 patients, which resulted in a decrease in the number of unique clones when additional repertoire features were included in the clonotyping definition (Figure 2B). Next, using Platypus, we were able to detect and visualize clonal expansion for both B and T cells (Figure 2C and D; Supplementary Figure S1A and B). We were able to relate isotype information with clonal expansion at single-cell resolution, thereby observing that the majority of the most expanded B-cell clones were predominantly of the IgA isotype and that some clones contained cells of multiple isotypes (e.g. BCRs with identical CDRH3 + CDRL3 sequence but different constant regions) (Figure 2D and Supplementary Figure S2B). We questioned how relaxing the clonotyping definition from identical CDRH3 + CDRL3 nucleotide sequence to identical V and J germline genes, identical CDR3 lengths, and a 70% CDR3 homology threshold would alter the clonal expansion profile for each patient. This analysis revealed that the clonal frequency and isotype distribution was minorly impacted for the most expanded clones for both patients (Supplementary Figure S1B). We next used built-in functions of Platypus to extract other common immune repertoire statistics and features, such as CDR3 length distribution and common sequence space motifs (sequence logo plots) (Figure 2E and F; Supplementary Figure S1C). This revealed tremendous diversity in the B-cell response at the most common paired CDRH3 + CDRL3 amino acid sequence length in both COVID-19 patients (Figure 2F). While we focused on the most frequent CDRH3 + CDRL3 sequence length, such an analysis using Platypus could theoretically be applied to other single-cell subsets, such as B cells with known antigen-specificity or cells that underwent extensive somatic hypermutation.

### Expanded clones demonstrate diverse germline gene usage

**Figure 3.**
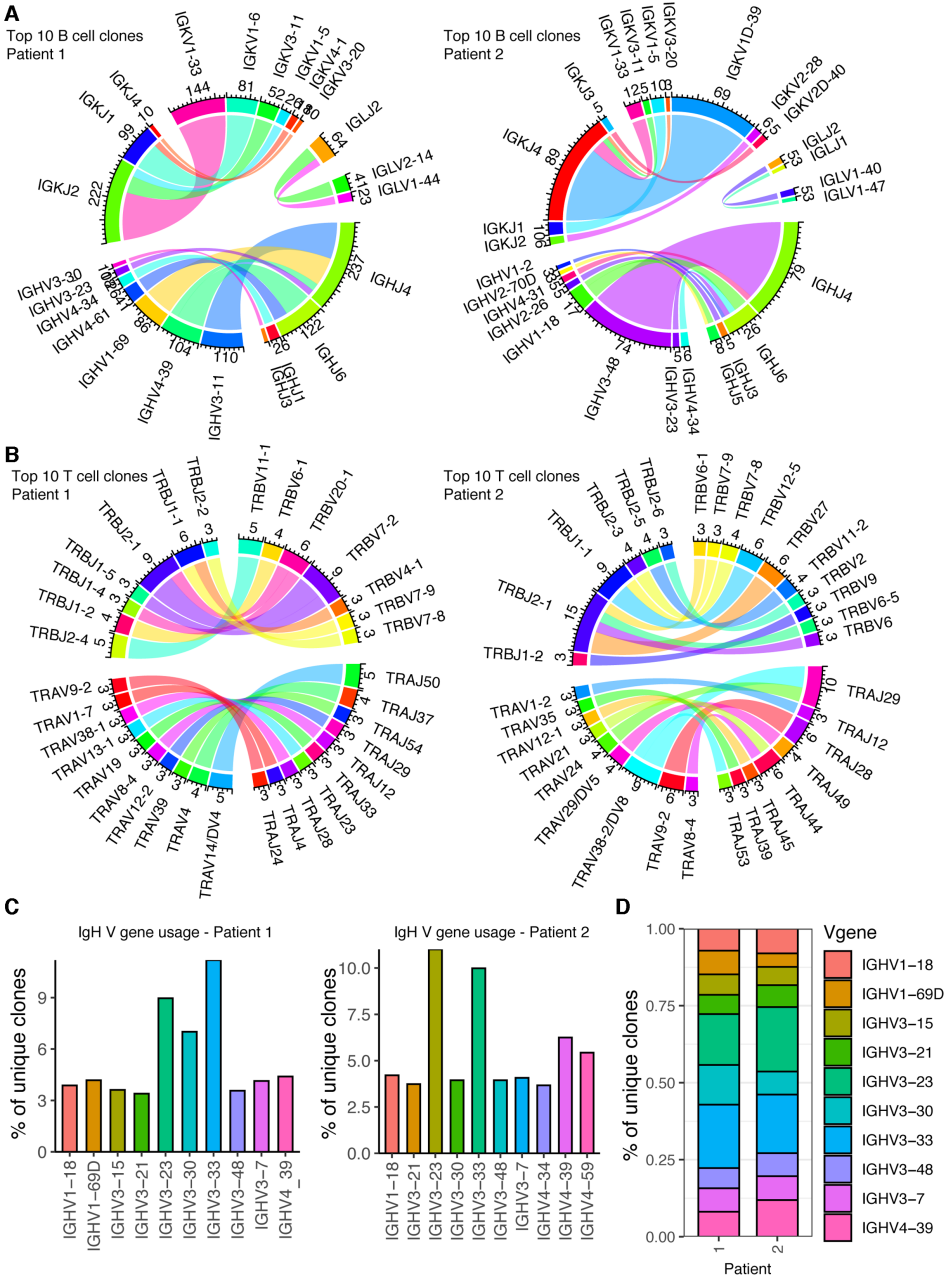
Analyzing and visualizing germline gene usage from B and T cell repertoires of two patients recently infected with SARS-CoV-2. (**A** and**B**) Circos plots for the ten most expanded B- and T-cell clones for each patient. Each line indicates the V and J gene usage for either the heavy or light chain (alpha/beta in the case of T cells) for an individual cell. The inner track number and the corresponding thickness of the bar indicates the number of cells using a given germline gene. Color corresponds to germline gene. Plots were produced using VDJ_VJ_usage_circos in Platypus. (**C**) V_H_ gene usage for the ten most used V_H_ genes in each repertoire. Clone is defined as unique CDRH3 + CDRL3 nucleotide sequence for B cells and unique CDRβ3 + CDRα3 nucleotide sequence for T cells. Plots were produced using VDJ_Vgene_usage in Platypus. (**D**) Stacked bar plot comparing the ten most used V_H_ genes across both patients. Plots produced using VDJ_Vgene_usage_stacked_barplot in Platypus.

Studying germline gene usage in the context of immune repertoires has been crucial to understanding selection in the context of disease, infection and immunization (26–28). We therefore designed Platypus to provide a diverse set of functions to quantify and visualize germline gene usage. Leveraging this pipeline for the two COVID-19 patients demonstrated diverse germline gene usage for both B and T cells, with certain V-J pairing arising more frequently than others across heavy, light, beta and alpha chains (Figure 3A and B; Supplementary Figures S2A–C). We could similarly investigate the most frequently used germline genes for each patient, revealing that while some genes were among the ten most expressed V genes in both patients (IgHV1–18, IgHV3–23), others (IgHV1–69D) were found exclusively in a single patient (Figure 3C). When integrating the V genes from both patients; however, the overall IgH V gene usage of the most expressed V genes was highly comparable (Figure 3D), potentially representing either SARS-CoV-2-induced clonal convergence or simply a representation of the germline gene usage of the natural human repertoire.

### Integration of single-cell immune repertoires and transcriptomes

**Figure 4.**
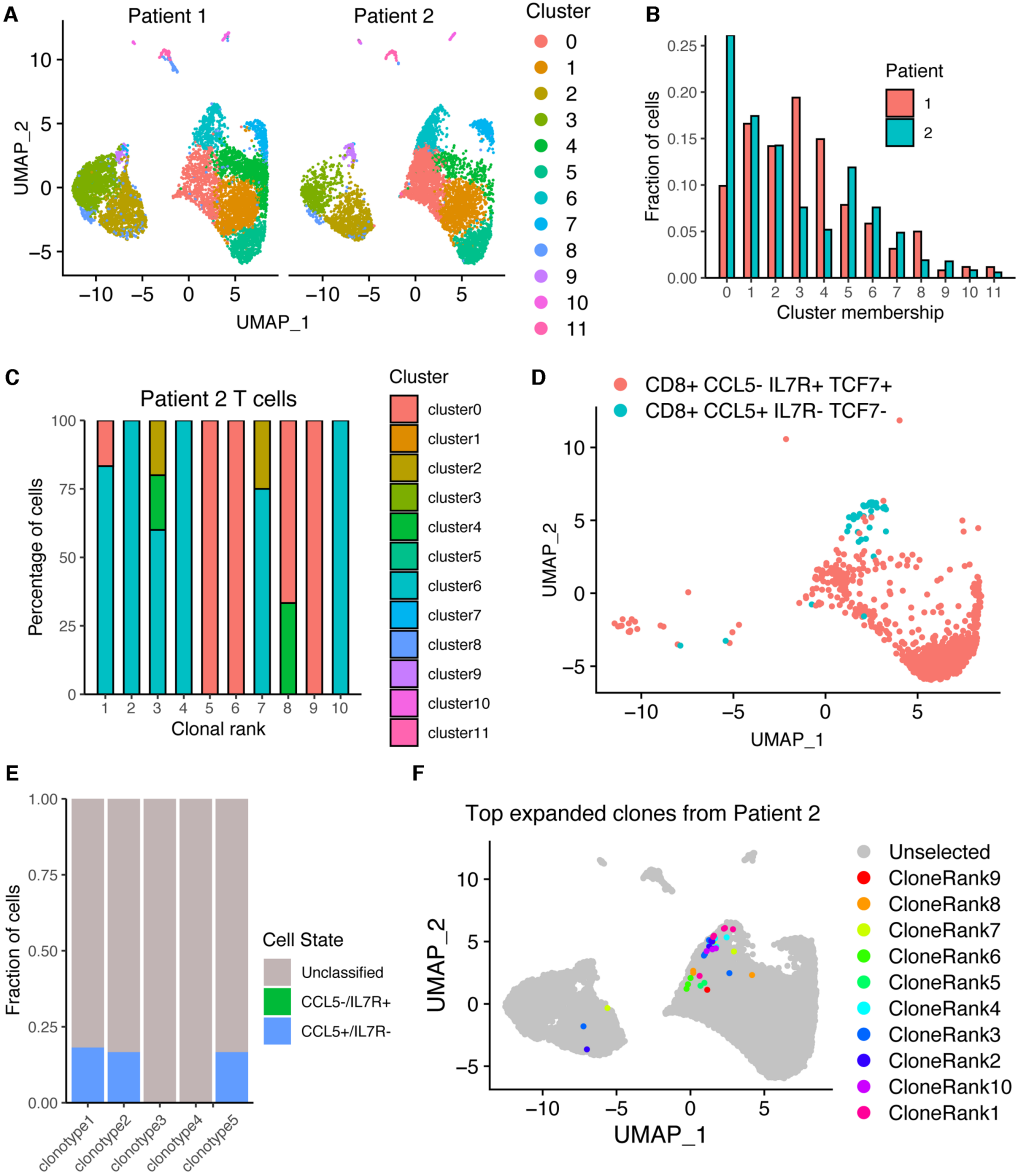
Integration of gene expression (GEX) and repertoire (VDJ) sequencing datasets from two patients recently infected with SARS-CoV-2. (**A**) Uniform manifold approximation projection (UMAP) of gene expression data from both COVID-19 patients. Cluster corresponds to the transcriptional clustering performed on the GEX datasets after excluding TCR and BCR receptor genes. Each point corresponds to a cell in one of the two patients. (**B**) Distribution of the fraction of cells located in each transcriptional cluster for all cells of each patient. Produced using the GEX_cluster_membership function in Platypus. (**C**) Distribution of the fraction of cells located in each transcriptional cluster for the top 10 most expanded T-cell clones of a single COVID-19 patient. Only those cells found in both GEX and VDJ sequencing datasets were included in the quantification. T-cell clone was defined by unique CDRβ3 + CDRα3 nucleotide sequence. (**D**) UMAP displaying those cells from both patients corresponding to memory-like or effector-like phenotypes labeled using the GEX_phenotype function from Platypus. (**E**) Fraction of cells from the top five most expanded T-cell clones from patient 2 matching either of the phenotypes in (D). Unclassified indicates lack of *CD8*, *CCL5*, *IL7R* and *TCF7* expression. Plot was produced with GEX_phenotype_per_clone in Platypus. (**F**) The 10 most expanded T-cell clones defined by unique CDRβ3 + CDRα3 nucleotide sequence from a single COVID-19 patient are highlighted on the UMAP containing all cells from both patients. Each point corresponds to a unique cell barcode. Only those cells found in both GEX and VDJ sequencing datasets were included in the quantification. Plot was produced using GEX_visualize_clones in Platypus.

A critical feature of Platypus is that it can seamlessly integrate single-cell immune repertoire data with transcriptome sequencing data. It allows users to directly interact with the commonly used scSeq transcriptome analysis program Seurat (16), while tuning parameters specifically relevant for immune repertoires. Therefore, we next investigated additional repertoire and transcriptional data of highly expanded B and T cell clonal groups, which allows us to relate repertoire information (e.g. expansion, CDR3 sequence and isotype) to phenotypic cellular behavior (e.g. whether a cell is proliferating, differentiated, activated, exhausted, etc.). We first integrated the transcriptome sequencing data from both COVID-19 patients by normalizing and scaling the data using default parameters in Seurat (although Platypus supports other normalization methods, such as SCTransform and Harmony) (16,17). We could thereby compute clusters based on gene expression and subsequently visualize the cells from each patient on the same 2D uniform manifold approximation projection (UMAP) plot (Figure 4A). Quantifying the distribution of cells in each cluster demonstrated variability between the two patients despite identical experimental conditions (Figure 4B). Utilizing the Seurat-based pipeline in Platypus, we performed global differential gene expression between the two patients and produced heatmaps of the most up- and downregulated genes based on either expression (log-fold change) or significance (adjusted *P*-value) (Supplementary Figure S3A). This revealed that varying expression levels of MHC-related and immune-related (*CXCR4*, *IL32*, *IL7R*) genes contributed to sample heterogeneity (Supplementary Figure S3A). To better characterize the gene expression signatures dictating the unsupervised clustering, we computed differentially expressed genes based on Seurat's FindAllMarkers function. A notable difference in the Platypus workflow is that the user can directly filter out mitochondrial and ribosomal genes and can additionally visualize either the most expressed (log fold-change) or most significant (adjusted *P*-value) genes that define each cluster. Performing this analysis for the COVID-19 patients demonstrated clear B- and T-cell clusters, defined by expression of *TCF7, LEF1* (clusters 0 and 1), *CD74, CD79A* (clusters 2 and 3), *IL7R* (cluster 4) and *CCL5, NKG7, GNLY* (cluster 6) (Supplementary Figure S3B). This was confirmed by highlighting gene expression of *CD4*, *CD8A*, *CD3E* and *CD19* on the UMAP, which revealed a separation between B and T cells and confirmed similarly distributed lymphocyte populations arising from both patients (Supplementary Figure S4). Of note is that we detected *CD3E* expression in the B-cell clusters on the UMAP, in addition to minor *CD19* expression in the T-cell clusters (Supplementary Figure S4), together suggesting the possibility of doublets in which B and T cells were present in the same microfluidic droplet.

We next investigated which transcriptional-based cluster contained the most expanded T-cell clones. We could demonstrate in one of the COVID-19 patients that some of the most expanded T-cell clones were located across multiple transcriptional clusters, demonstrating heterogeneous gene expression signatures (Figure 4C). Furthermore, we observed that the majority of expanded CD8+ T cells were located in cluster 6 (Figure 4C), which corresponded to high expression of *CCL5, NKG7* and *GNLY* (Supplementary Figure S1B). We next leveraged the GEX_phenotype function in Platypus to assign custom phenotypes based on gene expression to gain insight into the relationship between gene expression and clonal expansion. To this end, we labeled cells either as memory-like (*CD8+, CD44+*, *SELL+*, *IL7R+*, *CCL5-*) or effector-like (*CD8+, CD44+*, *SELL-*, *IL7R-*, *CCL5+*) (Figure 4D). *CCL5* was selected because it coincided with granzyme, *NKG7* and *GNLY* expression but was present in a higher proportion of cells (Supplementary Figure S3B). Quantifying the proportion of cells in the five most expanded T-cell clones indeed confirmed that they were more often labeled effector-like despite many more total cells corresponding to the memory-like phenotype (Figure 4E). This analysis further revealed that many of the cells from the most expanded clones were unclassified, which was likely due to relatively low RNA counts common to scSeq experiments. We therefore utilized the GEX_visualize_clones function in Platypus to overlay clonal information directly onto the UMAP plot to overcome the high proportion of cells lacking a phenotypic label. To investigate the transcriptional heterogeneity of clonally expanded T cells, we supplied the clonal index of the top 10 most expanded clones from a single patient and visualized where these cells lie within the UMAP plot, again revealing that many of the expanded clones were located in cluster 6 (Figure 4F). This approach can be leveraged to profile transcriptome signatures of well-defined clones, for example, ones in which antigen specificity is known.

**Figure 5.**
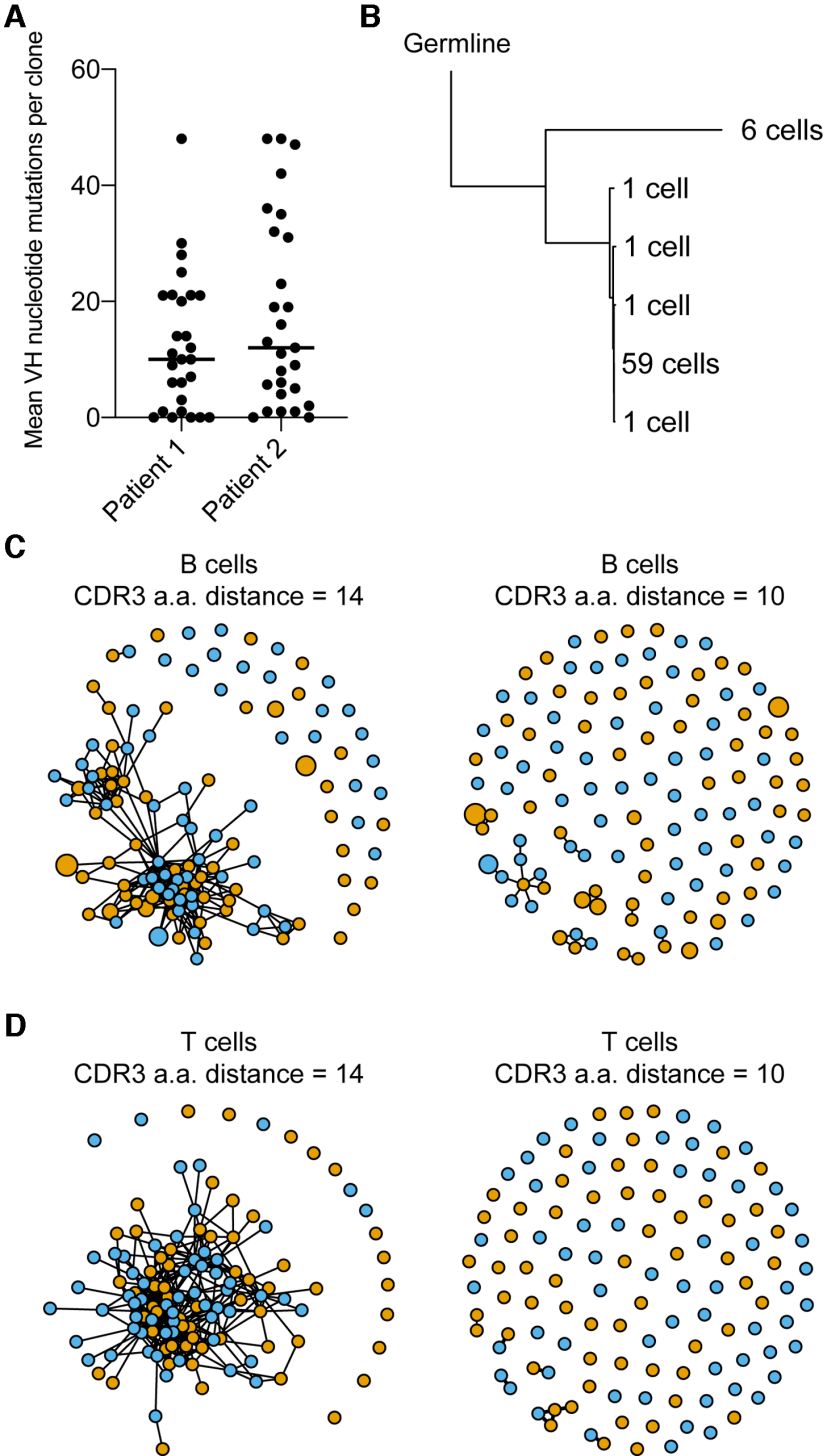
Functions from Platypus extract parameters relevant to the discovery of antigen-specific B- and T-cell clones. (**A**) Mean nucleotide somatic hypermutation for the 30 more expanded B-cell clones found in the blood repertoire following SARS-CoV-2 infection. Somatic hypermutation was quantified in the V and J segments for both the heavy and light chains for each cell by comparing misalignments to the reference germline segments. (**B**) Phylogenetic tree rooted by germline reference sequence. The reference germline as determined by cellranger was set as the root. The number of cells in the tip label corresponds to the number of unique nucleotide variants producing the exact, full-length antibody sequence. Each tip label represents a single unique nucleotide paired V_H_ + V_L_ sequence. (**C** and**D**) Similarity networks depicting the B- and T-cell clones that are separated by either <10 or 14 amino acid mutations in either CDRH3 or CDRL3 sequence (CDRβ3 + CDRα3 for T cells). Vertices represent unique clones defined as those cells containing identical CDHR3 + CDRL3 sequence (CDRβ3 + CDRα3 for T cells). Vertex colors represent each patient (patient 1 = orange, patient 2 = blue). Vertex size corresponds to the relative number of cells supporting each individual clone. Edges represent those clones that are separated by an edit distance of less than either 10 or 14 amino acid mutations in their CDR3 regions. Produced using VDJ_network in Platypus, which uses functions in igraph.

Next, we determined whether we could use Platypus to identify potentially virus-reactive B- and T-cell clones by integrating repertoire metrics, such as somatic hypermutation and clonal similarity with phenotypic markers of activation and differentiation. We first noticed that 30 most expanded B-cell clones had undergone somatic hypermutation in their V_H_ segments, and since these patients were convalescent but still actively infected, highly mutated antibodies represent potential-specific clones to SARS-CoV-2 (Figure 5A). Next, we used Platypus to infer the phylogenetic tree for the most expanded B-cell clone while also annotating information about clonal expansion (based on identified number of cell barcodes) (Figure 5B). Surprisingly, we uncovered that 59 cells produced the exact same, full-length nucleotide antibody sequence and was actually the least mutated relative to the unmutated germline ancestral sequence (Figure 5B). The potential specificity of an antibody that has minor somatic hypermutation is compatible with the recent discovery that B cells from COVID-19 patients that have germline-like antibodies with specificity to SARS-CoV-2 (22).

We next questioned whether we could detect similar CDR3 sequences shared between the two COVID-19 patient repertoires by inferring sequence similarity networks for the top 60 most expanded B- and T-cell clones (1). This involved first calculating the edit distance between the CDR3s of clones from both patients and then drawing edges between those clones that were separated by either <10 or 14 amino acid mutations in either the CDRH3 or the CDRL3 (or CDRβ3 + CDRα3 in T cells). We demonstrated two different thresholds here for illustration purposes, but this can be customized by the user. Our analysis revealed that the B-cell repertoires formed more clusters connected across patients than the T-cell repertoires when the more stringent amino acid threshold was applied (Figure 5C and D), thereby suggesting convergence of antibody sequences which may be a result of specificity to SARS-CoV-2. Highly similar, stereotypical antibody sequences from the memory B cells of different COVID-19 patients have recently been shown to occur (29). While these expanded, overlapping sequences were not present in a public database of known SARS-CoV-2 binders (CoVAbDb) (30), we did discover that an unexpanded B-cell clone not included in this network analysis had an identical CDRH3 (CARDLYYYGMDVW) to a known SARS-CoV-2-specific sequence. The lack of connected T-cell clones may be expected since it is likely these patients were not HLA-matched, in addition to the small portion of the blood repertoire sampled when performing single-cell sequencing.

**Figure 6.**
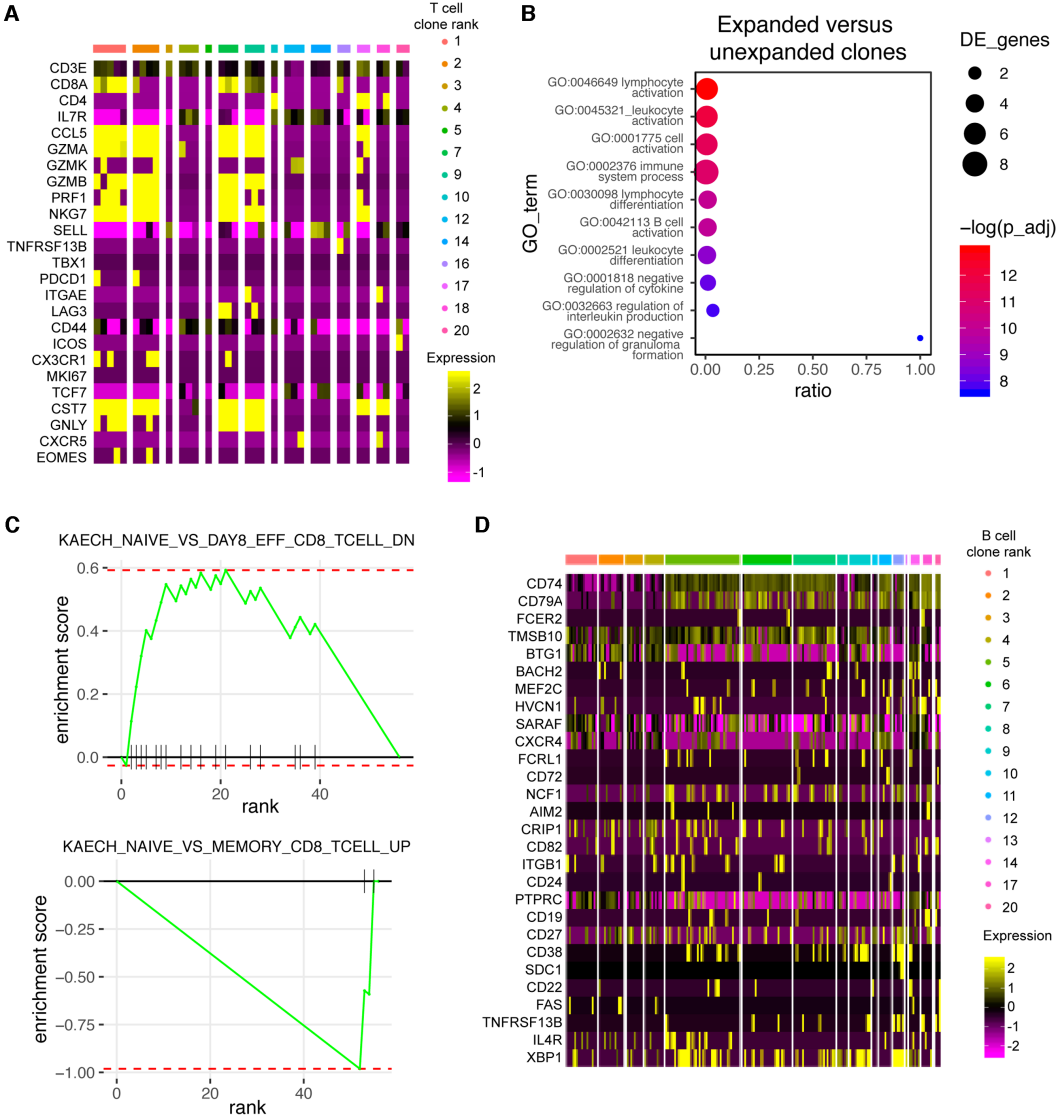
(**A**) Heatmap depicting normalized gene expression for the most expanded T-cell clones that were found in both the VDJ and gene expression (GEX) sequencing libraries from the blood repertoires of COVID-19 patients. The color of each column corresponds to an individual clonal family and the width of the bar corresponds to the number of cells found in the GEX library for that clone. Clone is defined as cells containing identical CDRβ3 + CDRα3 nucleotide sequences. (**B**) Gene ontology (GO) term enrichment for the top 10 most upregulated genes in the expanded clones relative to the unexpanded clones by average log fold-change. The color of each dot corresponds to adjusted *P*-value. The size of the dot corresponds to the number of genes of the particular GO term. Ratio corresponds to the number of differentially expressed genes relative to the number of total genes corresponding to each GO term. Produced using the GEX_GOterm function in Platypus. (**C**) Gene set enrichment (GSEA) plots based on the C7 immunological signatures from the Broad institute. The top 10 most upregulated (top panel) and the top 10 most downregulated (bottom panel) genes between expanded and unexpanded T-cell clones were produced using the GEX_GSEA function in Platypus. The enrichment plot of the highest scoring gene set is displayed. (**D**) Heatmap depicting normalized gene expression for the most expanded B cells found in both VDJ and GEX sequencing libraries. Clone is defined as those cells containing identical CDHR3 + CDRL3 sequence.

We lastly investigated whether signatures of activation or differentiation would reveal potential T- and B-cell clones that may have recently interacted with viral antigen. Platypus enables the user to relate clonotype information to user-defined transcriptional signatures, thereby connecting phenotypes involving antibody secretion, T-cell exhaustion, among others to immune receptor sequence. Using T and B cell-specific gene sets contained internally in Platypus, we could investigate phenotypic and differentiation (ontogeny) markers for expanded clones (Figure 6). Despite the low levels of T-cell clonal expansion in the blood repertoires, we nevertheless questioned if cells belonging to the same clonal family demonstrated transcriptional heterogeneity based on commonly used T-cell markers. Using the built-in gene sets internal to Platypus, we could visualize expression levels for genes, such as *CD44*, *PD1* (*PDCD1*), *LAG3*, *TCF7*, granzymes, perforin, *TBET*, *EOMES*, among others, at single-cell resolution (Figure 6A). Together, these genes allowed us to distinguish between naïve (*CD44*-, *TCF1*+, *SELL*+), memory (*IL7R*+), effector (*KLRG1*+) and exhausted (*PD1*+, *LAG3*+), subtypes that coexist within clonotypes (Figure 6A). Of interest was that the cells arising from expanded clones (e.g. clonotypes 1, 2, 3) expressed *CCL5* and granzymes, whereas several clones corresponding to either one or two cells did not express these markers (Figure 6A). Importantly, the user can customize the genes of interest to explore in the context of expanded clones. We next explicitly questioned whether gene ontology and gene set enrichment analyses would confirm our observations that expanded T-cell clones have a more effector-like phenotype. To this end, we computed and visualized the differentially expressed genes upregulated in the expanded (>1 cell) clones relative to the unexpanded (1 cell) clones, which confirmed that granzymes, *CCL5* and *NKG7* were among the most significantly upregulated genes by adjusted *P*-value. Leveraging the GEX_GOterm and GEX_GSEA functions in Platypus, we used these upregulated genes as input to unbiased gene ontology and gene set enrichment, which both resulted with hits relating to immune activation (Figure 6B) and enrichment matching effector T cells (i.e. the most enriched gene set using the upregulated genes from our clonally expanded cells matched a set containing downregulated genes in naïve T cells relative to effector T cells) (Figure 6C).

In the case of B cells, our analysis included the following markers: *B220* (*PTPRC*), *MS4A1*, *CD27*, *CD38* and *CD138* (*SDC1*), which distinguish between naïve, memory and plasma cells subsets (Figure 6D). This analysis further supported a transcriptional heterogeneity within the most expanded clones, as demonstrated by individual cells expressing varying degrees of B cell defining markers, such as *CD38*, *XBP1*, *CXCR4* and *TMSB10*. Clones expressing high levels of such activation markers (*CD38*) or genes associated with plasma cell differentiation (*XBP1*) are potentially interesting candidates for virus-specific antibodies (31). Taken together, Platypus enabled us to investigate and visualize repertoire and transcriptome parameters that can help facilitate the rapid discovery of relevant B- and T-cell clones.

## DISCUSSION

As in many areas of biology, researchers in adaptive immunity are poised to heavily utilize scSeq as an essential method to address basic and translational questions, therefore creating a substantial need for suitable bioinformatics tools. We have demonstrated here that Platypus enables rapid extraction, integration and analysis of scSeq of lymphocyte repertoires and transcriptomes to uncover meaningful insight on lymphocyte immunobiology and function. By using convalescent COVID-19 patients as an example, we were able to detect clonally expanded B and T cells at the single-cell resolution from <10 000 cells, suggesting these clones are present at high-levels in the remainder of the blood repertoire. Interestingly, we observed many expanded clones were of the IgA isotype and often did not coexist with other isotypes, highlighting that IgA antibodies may play an important role in the response to SARS-CoV-2. We could relate these expanded clones to diverse cellular phenotypes based on district transcriptional clusters. This revealed that even when cells belonged to the same BCR or TCR clonal group, they could still be found in different transcriptional clusters. This highlights that lymphocytes sharing the same immune receptor specificity may still undergo very different cell fates and functions in the course of an immune response. We could furthermore analyze various repertoire parameters including somatic hypermutation, clonal evolution and clonal convergence at the single-cell resolution. Platypus allowed us to analyze and extract full-length, paired, heavy-light chain information for clonally expanded and heavily mutated clones, which offers a possible approach to identify SARS-CoV-2-specific lymphocytes. Leveraging Platypus, we were able to visualize this information and have full-length BCR and TCR sequences ready for cloning in less than 10 lines of code—something that can greatly accelerate the discovery of adaptive immune therapeutics. In conclusion, Platypus enables a broad range of immunologists and bioinformaticians alike to gain quantitative insight at a single-cell resolution of how immune repertoire parameters relate to heterogeneous transcriptome information.

## DATA AVAILABILITY

The R package, code and example data used in this publication are available at github.com/alexyermanos/Platypus and https://doi.org/10.5281/zenodo.4140161.

## Supplementary Material

lqab023_Supplemental_File

## References

[1] Miho E., Yermanos A., Weber C.R., Berger C.T., Reddy S.T., Greiff V. Computational strategies for dissecting the high-dimensional complexity of adaptive immune repertoires. Front. Immunol. 2018; 9:224. 29515569 10.3389/fimmu.2018.00224 PMC5826328

[2] Greiff V., Miho E., Menzel U., Reddy S.T. Bioinformatic and statistical analysis of adaptive immune repertoires. Trends Immunol. 2015; 36:738–749. 26508293 10.1016/j.it.2015.09.006

[3] Georgiou G., Ippolito G.C., Beausang J., Busse C.E., Wardemann H., Quake S.R. The promise and challenge of high-throughput sequencing of the antibody repertoire. Nat. Biotech. 2014; 32:158–168. 10.1038/nbt.2782 PMC4113560 24441474

[4] Yaari G., Kleinstein S.H. Practical guidelines for B-cell receptor repertoire sequencing analysis. Genome Med. 2015; 7:121. 26589402 10.1186/s13073-015-0243-2 PMC4654805

[5] Shugay M., Britanova O.V., Merzlyak E.M., Turchaninova M.A., Mamedov I.Z., Tuganbaev T.R., Bolotin D.A., Staroverov D.B., Putintseva E.V., Plevova K. et al. Towards error-free profiling of immune repertoires. Nat. Meth. 2014; 11:653–655. 10.1038/nmeth.2960 24793455

[6] Brown A.J., Snapkov I., Akbar R., Pavlović M., Miho E., Sandve G.K., Greiff V. Augmenting adaptive immunity: progress and challenges in the quantitative engineering and analysis of adaptive immune receptor repertoires. Mol. Syst. Des. Eng. 2019; 4:701–736.

[7] Picelli S., Faridani O.R., Björklund Å.K., Winberg G., Sagasser S., Sandberg R. Full-length RNA-seq from single cells using Smart-seq2. Nat. Protoc. 2014; 9:171–181. 24385147 10.1038/nprot.2014.006

[8] Horns F., Dekker C.L., Quake S.R. Memory B-cell activation, broad anti-influenza antibodies, and bystander activation revealed by single-cell transcriptomics. Cell Rep. 2020; 30:905–913. 31968262 10.1016/j.celrep.2019.12.063 PMC7891556

[9] Gate D., Saligrama N., Leventhal O., Yang A.C., Unger M.S., Middeldorp J., Chen K., Lehallier B., Channappa D., De Los Santos M.B. et al. Clonally expanded CD8 T cells patrol the cerebrospinal fluid in Alzheimer's disease. Nature. 2020; 577:399–404. 31915375 10.1038/s41586-019-1895-7 PMC7445078

[10] Singh M., Al-Eryani G., Carswell S., Ferguson J.M., Blackburn J., Barton K., Roden D., Luciani F., Giang Phan T., Junankar S. et al. High-throughput targeted long-read single cell sequencing reveals the clonal and transcriptional landscape of lymphocytes. Nat. Commun. 2019; 10:3120. 31311926 10.1038/s41467-019-11049-4 PMC6635368

[11] Meyer K.D. DART-seq: an antibody-free method for global m6A detection. Nat. Methods. 2019; 16:1275–1280. 31548708 10.1038/s41592-019-0570-0 PMC6884681

[12] Wu T.D., Madireddi S., de Almeida P.E., Banchereau R., Chen Y.-J.J., Chitre A.S., Chiang E.Y., Iftikhar H., O’Gorman W.E., Au-Yeung A. et al. Peripheral T-cell expansion predicts tumour infiltration and clinical response. Nature. 2020; 579:274–278. 32103181 10.1038/s41586-020-2056-8

[13] Bossel Ben-Moshe N., Hen-Avivi S., Levitin N., Yehezkel D., Oosting M., Joosten L.A.B., Netea M.G., Avraham R. Predicting bacterial infection outcomes using single cell RNA-sequencing analysis of human immune cells. Nat. Commun. 2019; 10:3266. 31332193 10.1038/s41467-019-11257-y PMC6646406

[14] Cheung D., Adams B.A., McDonnell W.J., Jaffe D.B., Puleo A.R., Sukovich D.J., Reyes D.J., Royall A.J., Chi J.C., Srinavas N.J. et al. Profiling the immune infiltrate in tumor samples at single cell resolution. J. Immunol. 2020; 204:243.20.31907265

[15] King H.W., Orban N., Riches J.C., Clear A.J., Warnes G., Teichmann S.A., James L.K. Single-cell analysis of human B cell maturation predicts how antibody class switching shapes selection dynamics. Sci Immunol. 2021; 6:eabe6291. 33579751 10.1126/sciimmunol.abe6291

[16] Butler A., Hoffman P., Smibert P., Papalexi E., Satija R. Integrating single-cell transcriptomic data across different conditions, technologies, and species. Nat. Biotechnol. 2018; 36:411–420. 29608179 10.1038/nbt.4096 PMC6700744

[17] Korsunsky I., Millard N., Fan J., Slowikowski K., Zhang F., Wei K., Baglaenko Y., Brenner M., Loh P., Raychaudhuri S. Fast, sensitive and accurate integration of single-cell data with Harmony. Nat. Methods. 2019; 16:1289–1296. 31740819 10.1038/s41592-019-0619-0 PMC6884693

[18] Zhang X., Xu C., Yosef N. Simulating multiple faceted variability in single cell RNA sequencing. Nat. Commun. 2019; 10:2611. 31197158 10.1038/s41467-019-10500-w PMC6565723

[19] Zappia L., Phipson B., Oshlack A. Splatter: simulation of single-cell RNA sequencing data. Genome Biol. 2017; 18:174. 28899397 10.1186/s13059-017-1305-0 PMC5596896

[20] Lefranc M.-P., Giudicelli V., Duroux P., Jabado-Michaloud J., Folch G., Aouinti S., Carillon E., Duvergey H., Houles A., Paysan-Lafosse T. et al. IMGT^®^, the international ImMunoGeneTics information system^®^ 25 years on. Nucleic Acids Res. 2014; 43:413–422. 10.1093/nar/gku1056 PMC4383898 25378316

[21] Li S., Lefranc M.-P., Miles J.J., Alamyar E., Giudicelli V., Duroux P., Freeman J.D., Corbin V.D.A., Scheerlinck J.-P., Frohman M.A. et al. IMGT/HighV QUEST paradigm for T-cell receptor IMGT clonotype diversity and next generation repertoire immunoprofiling. Nat. Commun. 2013; 4:2333. 23995877 10.1038/ncomms3333 PMC3778833

[22] Bolotin D.A., Poslavsky S., Mitrophanov I., Shugay M., Mamedov I.Z., Putintseva E.V., Chudakov D.M. MiXCR: software for comprehensive adaptive immunity profiling. Nat. Meth. 2015; 12:380–381. 10.1038/nmeth.3364 25924071

[23] Rosenberg A.B., Roco C.M., Muscat R.A., Kuchina A., Sample P., Yao Z., Graybuck L.T., Peeler D.J., Mukherjee S., Chen W. et al. Single-cell profiling of the developing mouse brain and spinal cord with split-pool barcoding. Science. 2018; 360:176–182. 29545511 10.1126/science.aam8999 PMC7643870

[24] Yermanos A., Greiff V., Krautler N.J., Menzel U., Dounas A., Miho E., Oxenius A., Stadler T., Reddy S.T. Comparison of methods for phylogenetic B-cell lineage inference using time-resolved antibody repertoire simulations (AbSim). Bioinformatics. 2017; 33:3938–3946. 28968873 10.1093/bioinformatics/btx533

[25] Yermanos A.D., Dounas A.K., Stadler T., Oxenius A., Reddy S.T. Tracing antibody repertoire evolution by systems phylogeny. Front. Immunol. 2018; 9:2149. 30333820 10.3389/fimmu.2018.02149 PMC6176079

[26] Greiff V., Menzel U., Miho E., Weber C., Riedel R., Cook S., Valai A., Lopes T., Radbruch A., Winkler T.H. et al. Systems analysis reveals high genetic and antigen-driven predetermination of antibody repertoires throughout B-cell development. Cell Rep. 2017; 19:1467–1478. 28514665 10.1016/j.celrep.2017.04.054

[27] Yermanos A., Sandu I., Pedrioli A., Borsa M., Wagen F., Oetiker N., Welten S.P.M., Pallmer K., Reddy S.T., Oxenius A. Profiling virus-specific Tcf1+ T-cell repertoires during acute and chronic viral infection. Front. Immunol. 2020; 11:986. 32547546 10.3389/fimmu.2020.00986 PMC7272574

[28] Kräutler N.J., Yermanos A., Pedrioli A., Welten S.P.M., Lorgé D., Greczmiel U., Bartsch I., Scheuermann J., Kiefer J.D., Eyer K. et al. Quantitative and qualitative analysis of humoral immunity reveals continued and personalized evolution in chronic viral infection. Cell Rep. 2020; 30:997–1012. 31995768 10.1016/j.celrep.2019.12.088

[29] Robbiani D.F., Gaebler C., Muecksch F., Lorenzi J.C.C., Wang Z., Cho A., Agudelo M., Barnes C.O., Gazumyan A., Finkin S. et al. Convergent antibody responses to SARS-CoV-2 in convalescent individuals. Nature. 2020; 584:437–442. 32555388 10.1038/s41586-020-2456-9 PMC7442695

[30] Raybould M.I.J., Kovaltsuk A., Marks C., Deane C.M. CoV-AbDab: the coronavirus antibody database. Bioinformatics. 2020; https://doi.org/10.1093/bioinformatics/btaa739. 10.1093/bioinformatics/btaa739 PMC7558925 32805021

[31] Todd D.J., McHeyzer-Williams L.J., Kowal C., Lee A.-H., Volpe B.T., Diamond B., McHeyzer-Williams M.G., Glimcher L.H. XBP1 governs late events in plasma cell differentiation and is not required for antigen-specific memory B-cell development. J. Exp. Med. 2009; 206:2151–2159. 19752183 10.1084/jem.20090738 PMC2757870

[32] Wagih O. ggseqlogo: a versatile R package for drawing sequence logos. Bioinformatics. 2017; 33:3645–3647. 29036507 10.1093/bioinformatics/btx469

[33] Gu Z., Gu L., Eils R., Schlesner M., Brors B. circlize Implements and enhances circular visualization in R. Bioinformatics. 2014; 30:2811–2812. 24930139 10.1093/bioinformatics/btu393

[34] Robinson M.D., McCarthy D.J., Smyth G.K. edgeR: a Bioconductor package for differential expression analysis of digital gene expression data. Bioinformatics. 2010; 26:139–140. 19910308 10.1093/bioinformatics/btp616 PMC2796818

[35] Ritchie M.E., Phipson B., Wu D., Hu Y., Law C.W., Shi W., Smyth G.K. limma powers differential expression analyses for RNA-sequencing and microarray studies. Nucleic Acids Res. 2015; 43:e47. 25605792 10.1093/nar/gkv007 PMC4402510

[36] Sergushichev A.A. An algorithm for fast preranked gene set enrichment analysis using cumulative statistic calculation. 2016; bioRxiv doi:20 June 2016, preprint: not peer reviewed 10.1101/060012.

[37] Liberzon A., Subramanian A., Pinchback R., Thorvaldsdóttir H., Tamayo P., Mesirov J.P. Molecular signatures database (MSigDB) 3.0. Bioinformatics. 2011; 27:1739–1740. 21546393 10.1093/bioinformatics/btr260 PMC3106198

