# Supplementary material for: Platypus: an open-access software for integrating lymphocyte single-cell immune repertoires with transcriptomes
Source: NAR Genom Bioinform. 2021 Apr 14;3(2):lqab023. doi: 10.1093/nargab/lqab023 (PMC8046018; doi:10.1093/nargab/lqab023)
Supplement: lqab023_Supplemental_File [file lqab023_supplemental_file.docx]

Supporting Information for:

Platypus: an open-access software for integrating lymphocyte single-cell immune repertoires with transcriptomes

Alexander Yermanos^1,2,3,^*, Andreas Agrafiotis^1^, Raphael Kuhn^1^, Damiano Robbiani^1^, Josephine Yates^1^, Chrysa Papadopoulou^1^, Jiami Han^1^, Ioana Sandu^2^, Cédric Weber^1^, Florian Bieberich^1^, Rodrigo Vazquez-Lombardi^1^, Andreas Dounas^4^, Daniel Neumeuer^1^, Annette Oxenius^2^, Sai T. Reddy^1,^*

^1^Department of Biosystems Science and Engineering, ETH Zurich, Basel, Switzerland

^2^Institute of Microbiology, ETH Zurich, Zurich, Switzerland

^3^Department of Pathology and Immunology, University of Geneva, Geneva, Switzerland

^4^Institute for Biomedical Engineering, University and ETH Zurich, Zurich, Switzerland

*correspondence: ayermanos@gmail.com; sai.reddy@ethz.ch

Figure S1. Additional B and T cell repertoire features from two convalescent COVID-19 patients. (A) Clonal expansion profiles of the T and B cells from the blood repertoires of one individual recently infected with SARS-CoV-2. Clone is defined as unique CDRβ3 + CDRα3 nucleotide sequence for T cells and CDRH3 + CDRL3 nucleotide sequence for B cells. (B) Clonotyping strategies involving CDR3 sequence identity and germline gene usage have a minor impact on clonal expansion profiles of the most expanded clones. (C) Length distribution of the paired CDRH3 + CDRL3 amino acid sequences from the B cell clones of a single patient.


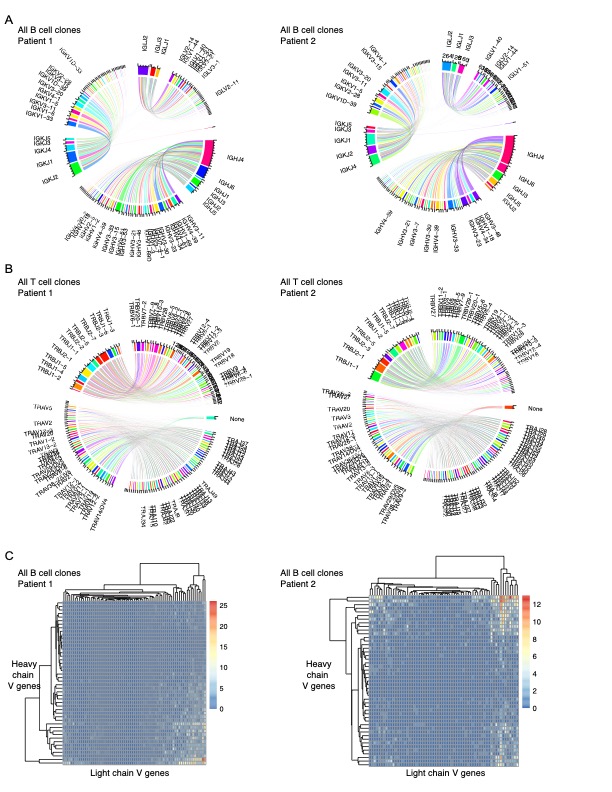


Figure S2. Germline gene usage for all B and T cell clones. (A-B) Circos plots for the ten most expanded B cell and T cell clones for each patient, respectively. Each line indicates the V and J gene usage for either the heavy or light chain (alpha/beta in the case of T cells). Color corresponds to germline gene. Plots were produced using VDJ_VJ_usage_circos in Platypus. (C) Heatmaps visualizing the paired heavy and light chain variable gene usage. Germline gene usage was extracted using VDJ_Vgene_usage in Platypus, which was subsequently used as input to the pheatmap function in the pheatmap package.

Figure S3. Differential gene expression analysis from two convalescent COVID-19 patients. (A) The top ten upregulated and top ten downregulated differentially expressed genes between the two patients, ranked by either average log fold change (left panel) or adjusted p value (right panel). Mitochondrial (MT) and ribosomal (RPS, RPL) genes were filtered before visualization. Heatmap produced using GEX_DEgenes_per_sample in Platypus, which is based on FindMarkers and DoHeatmap from Seurat. Identity 1 (red) corresponds to patient 1 and identity 2 (blue) corresponds to patient 2. (B) The top genes expressed in each cluster based on highest log fold-change compared to all other cells. Identity refers to transcriptional cluster and intensity corresponds to normalized expression. Mitochondrial (MT) and ribosomal (RPS, RPL) genes were filtered before visualization. Calculation, filtering, and visualization performed using GEX_cluster_genes and GEX_cluster_genes_heatmap in Platypus, which relies on FindAllMarkers and DoHeatmap from Seurat.


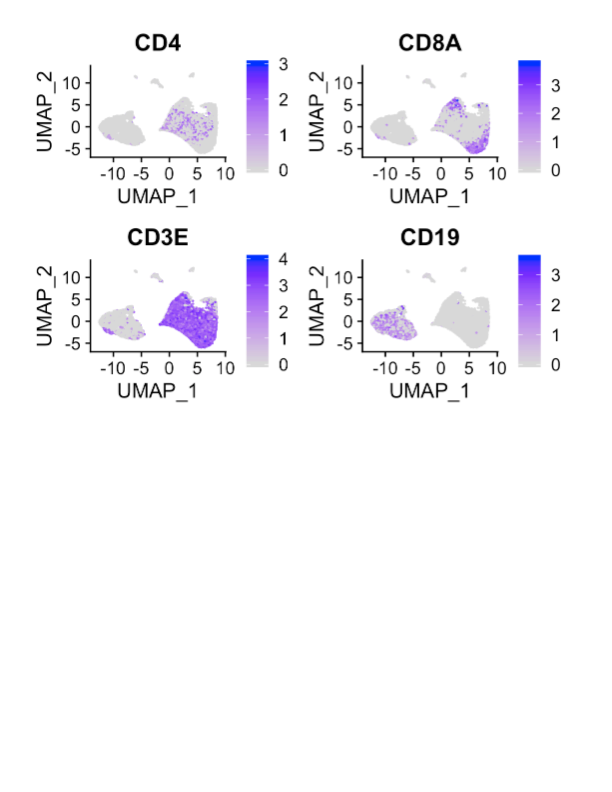


Figure S4. Uniform manifold approximation projection (UMAP) plot depicting the expression of *CD4*, *CD8A*, *CD3E*, and *CD19* to identify B and T cells.


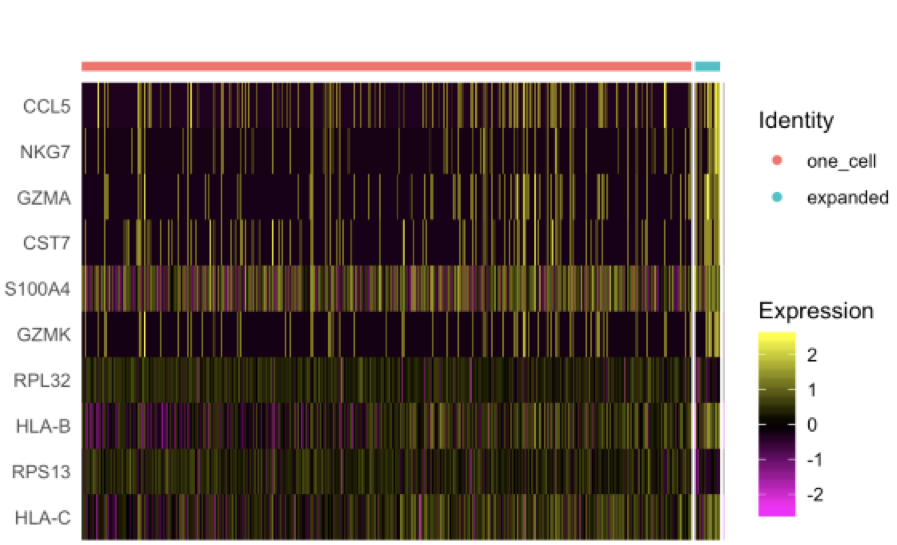


Figure S5. Top ten most upregulated genes by average log fold-change in the expanded T cell clones compared to the clones supported by only one cell. Intensity corresponds to normalized gene expression.
